# Creasote in Diarrhea

**Published:** 1851-10

**Authors:** 


*Creosote in Diarrhea.*—M. Kesteven extols the efficacy of creosote in the cure of diarrhea. The form in which he used it was: ℞.—Creosoti m. j to m. v; Spt. ammon. arom. m. xv to ʒ j; Aq. ℥ j to ℥ iss. Where pain has been severe, Tinc. camph. co. had been added.

In no single case, Mr. K. says, has creosote failed to be of signal benefit; in most cases one single dose was sufficient to arrest the course of the disease; in very few instances has it been requisite to administer more than the second dose.—*Amer. Jour. Med. Science.*

